# Testing the Interpersonal-Behavior model to explain intentions to use patient-delivered partner therapy

**DOI:** 10.1371/journal.pone.0233348

**Published:** 2020-05-20

**Authors:** Steven A. John, Jennifer L. Walsh, Katherine G. Quinn, Young Ik Cho, Lance S. Weinhardt

**Affiliations:** 1 Center for AIDS Intervention Research (CAIR), Department of Psychiatry and Behavioral Medicine, Medical College of Wisconsin, Milwaukee, Wisconsin, United States of America; 2 Joseph J. Zilber School of Public Health, University of Wisconsin-Milwaukee, Milwaukee, Wisconsin, United States of America; University Lyon 1 Faculty of Dental Medicine, FRANCE

## Abstract

**Background:**

Patient-delivered partner therapy (PDPT) is an evidence-based method of partner treatment, but further research was needed to understand theoretical underpinnings of potential PDPT use.

**Purpose:**

We sought to develop and test a theoretical framework to understand PDPT intentions.

**Methods:**

A Midwestern sample of sexually transmitted infection clinic patients were recruited to participate in a three-phase study incorporating semi-structured interviews (*n* = 20, total), cognitive interviews (*n* = 5), and surveys (*n* = 197; *M*_age_ = 31.3, 61% male, 91% Black or African-American). Thematic analysis was conducted to identify major themes, which guided development and testing of a theoretical framework on PDPT intentions using structural equation modeling.

**Results:**

We identified themes of information (knowledge); motivation (individual and partner protection beliefs, partner and provider motivation-to-comply); social support (sexual health and general); and behavioral skills (partner notification, medication delivery, and communication skills self-efficacy) in thematic analysis. The developed Interpersonal-Behavior model demonstrated good model fit in structural equation modeling [χ2(36) = 95.56, *p*<0.01; RMSEA = 0.09 (0.07–0.11, 90%C.I.); CFI = 0.94; SRMR = 0.05]. Information was associated with motivation (*β* = 0.37, *p*<0.001) and social support (*β* = 0.23, *p* = 0.002). Motivation was associated with social support (*β* = 0.64, *p*<0.001) and behavioral skills (*β* = 0.40, *p*<0.001), and social support was associated with behavioral skills (*β* = 0.23, *p* = 0.025). Behavioral skills were associated with higher PDPT intentions (*β* = 0.31, *p*<0.001), partially mediated the association of motivation with intentions (*β*_direct_ = 0.53, *p*<0.001; *β*_indirect_ = 0.12, 95%CI: 0.03–0.30), and fully mediated the association of social support with intentions (*β*_indirect_ = 0.07, 95%CI: 0.00–0.21).

**Conclusions:**

The Interpersonal-Behavior model seems appropriate for PDPT intentions but should be tested longitudinally with PDPT outcomes and other interpersonal health behaviors.

## Introduction

Sexually transmitted infections (STIs) continue to be a common morbidity in the United States (US). While incidence rates of HIV are declining because of biomedical treatment and prevention [1], the number of new infections of common bacterial STIs—such as chlamydia and gonorrhea—remains stagnantly high with indications of increasing prevalence [2]. Moreover, disparities in STI diagnoses by race/ethnicity are unwavering; Black and African Americans remain at five to ten times higher risk of common bacterial STIs compared to their White counterparts [2]. Black men and women are also 3.0 and 1.6 times more likely to experience repeat infection compared to non-Black patients, respectively [3]. Further efforts are needed to mitigate the current trends in bacterial STIs among US-based populations, particularly among those disproportionately affected.

Partner notification services are paramount to reducing STI transmission. Patient-delivered partner therapy (PDPT)—wherein a patient diagnosed with a bacterial STI is given extra medication to deliver to their sexual partners—is one promising mechanism to increase rates of partner treatment, reduce the risk of repeat infections among index patients, and decrease community prevalence [4, 5]. However, the 8% average repeat infection rate among patients provided PDPT in randomized controlled trials [4] indicates further efforts are needed to better prepare patients to give PDPT to their partners and reduce repeat infection risk. Researchers previously identified potential factors that—when aggregated across samples and context [6–15]—fit within constructs of the Information-Motivation-Behavioral skills model [16–19], a framework for understanding sexual health-related behaviors previously validated among STI clinic patients [20–23] and commonly used among many different populations for HIV and STI prevention [24–48]. Specifically, the Information-Motivation-Behavioral skills model posits that individuals must be highly informed, motivated, and behaviorally skilled to initiate HIV/STI prevention behaviors [16–19]. The Information-Motivation-Behavioral skills model serves as an appropriate starting point, but further examination was needed to determine whether the model needed to be adapted to better understand barriers and facilitators of potential PDPT use among STI clinic patients. In this mixed-method study, we sought to: 1) determine barriers and facilitators to PDPT use, 2) develop measurement tools with good construct validity and reliability, and 3) test a theoretical framework on intentions to use PDPT to support the development of a behavioral intervention.

## Methods

### Recruitment and procedures

Data for this analysis were taken from a mixed-method study of patients presenting for STI/HIV counseling and testing services at a large, publicly funded STI clinic in Milwaukee, Wisconsin, US. Milwaukee is an important city for STI prevention work, where population-adjusted rates of gonorrhea and chlamydia infections were ranked number 1 and 3 nationwide, respectively, among metropolitan areas in 2016 [2]. Inclusion criteria for participation was based on age only; patients 18 years of age or older were approached in the waiting room and invited to participate between March and June 2016. As described previously [49, 50], recruitment occurred in three phases by design and is outlined below. All study procedures were approved by the Institutional Review Board of the University of Wisconsin-Milwaukee.

### Ethical approval

All procedures performed in studies involving human participants were in accordance with the ethical standards of the institutional and/or national research committee and with the 1964 Helsinki declaration and its later amendments or comparable ethical standards.

### Informed consent

Informed consent was obtained from all individual participants included in the study.

#### Phase 1

In Phase 1, five patients—of 11 clinic patients approached to determine eligibility and invited to participate in the study—were interviewed individually using a semi-structured interview guide with open-ended questions developed based on themes identified within extant literature (i.e., information, attitudes, subjective norms, and behavioral skills). Individual interviews were used to explore participants’ perceptions within their personal context [51]. Interviewees were given a brief purpose statement about the interview, a scenario in which they were diagnosed with a bacterial STI (e.g., chlamydia) at their visit, and a description of having clinic nurses give them antibiotics to take to their sexual partners (i.e., PDPT). An example question from the interview guide is “What are some questions you might ask a healthcare provider before considering if you would deliver medications to your sexual partners?”

#### Phase 2

In Phase 2, five more patients—of 23 clinic patients approached by research staff—participated in one-on-one, highly-structured cognitive interviews. The main purpose of Phase 2 interviews was to determine the content validity of survey items based on respondents’ individual thought processes [52] developed based on Phase 1 interviews and extant literature. Participants were invited to openly discuss their opinions about the topics included in the survey items; these responses were used for qualitative analysis and modification of survey measures.

#### Phase 3

Finally, we conducted convenience sampling of STI clinic patients to participate in a one-time survey; 600 patients were approach to be screened for eligibility, of whom 200 consented to participate in a study with a standardized survey questionnaire developed based on the results of Phase 2. A purposive sampling strategy was then used to select a sample of 15 patients balanced in terms of sex, race, and quantitative ratings of PDPT acceptability. The purpose of the Phase 3 one-on-one, semi-structured interviews was to identify potential intervention mechanisms, barriers and facilitators to PDPT delivery, and other important information for planning a randomized controlled trial. Open-ended interview questions—using a semi-structured interview guide—were developed to elicit responses about the typical standard-of-care intervention mechanism of information only, ask questions about patients’ barriers and facilitators of potential PDPT use, and inquire about including a rapid HIV test with PDPT (not discussed here; see [50]). An example question from the interview guide was “What might stand in the way of your success in providing medications for you to give to your sexual partner(s)?”

### Measures

Survey measures were informed by thematic analysis of Phase 1 semi-structured interviews, refined to improve content validity after testing survey items in Phase 2 cognitive interviews, and empirically tested to determine unidimensional construct assessment and reliability. In addition to a demographic questionnaire, the final measures assessed major themes of information, motivational factors, social support, behavioral skills, and PDPT intentions.

#### Demographics

Participants were asked to report their age, sex assigned at birth, race/ethnicity, educational attainment, employment status, and health insurance status.

#### Information

A four-item scale was developed to assess partner treatment knowledge (PT-KQ-4; α = 0.77). After an overview of a hypothetical scenario where the participant was diagnosed with an STI, participants were asked to respond to the PT-KQ-4. An example item was “Your most recent unprotected sex partner could have the same infection as you even if they are not showing any signs or symptoms of an STI.” Response categories included true, false, and don’t know, which were coded into correct/incorrect with don’t know responses coded incorrect.

#### Motivational factors

All scales assessing motivational factors used five-point response categories ranging *very untrue* to *very true of what I believe*. A three-item scale was developed to assess individual protection beliefs (α = 0.62). An example item was “I can reduce my chance of getting another STI if my partner(s) get treated.” A five-item scale assessed protection beliefs for others (α = 0.79). An example item was “My partners’ health is important to me.” Motivation-to-comply with partner norms was assessed using a three-item scale (α = 0.73). An example item was “I would take medications to my partner(s) if I thought they would want me to.” Motivation-to-comply with healthcare provider norms was assessed using a 4-item scale (α = 0.81). An example item was “I would take medications to my partner(s) if my healthcare provider wanted me to.”

#### Social support

We assessed both general and sexual health social support. The six-item social support questionnaire was used to assess general social support (α = 0.95) [53]. An example item was “I have someone who I can really count on to be dependable when I need help.” A three-item sexual health social support scale was developed and used (α = 0.88). An example item was “I have someone who I can talk to about sexual health issues.” Five-point response categories ranging *very untrue* to *very true of what I believe* were used for both social support scales.

#### Behavioral skills

Three scales were developed to assess self-efficacy constructs of behavioral skills, each of which used five-point response categories ranging *not at all confident* to *extremely confident*. Partner notification self-efficacy was assessed using a six-item scale (α = 0.87). An example item was “How confident are you in your ability to tell your partner(s) of an STI.” Medication delivery self-efficacy was assessed using a four-item scale (α = 0.81). An example item was “How confident are you in your ability to delivery medications to your partner(s) for treatment?” Communication skill self-efficacy was assessed using a 6-item scale (α = 0.89). An example item was “How confident are you in your ability to communicate with your partner(s) about sexual health issues?”

#### PDPT intentions

A six-item scale assessed behavioral intentions of STI partner notification and PDPT use for all, main, and non-main partners (α = 0.89). An example item was “Imagine you were diagnosed with an STI today. [State whether the following are true or not of what you believe:] I would definitely give my main recent sex partners (last 3 months) medications for treatment if provided from my healthcare provider.” Five-point response categories ranged *very untrue* to *very true of what I believe*.

### Data analysis

Qualitative data were analyzed using a thematic analysis [54] to guide and provide context to the quantitative analysis. Data from all three phases were transcribed verbatim before undergoing an aggregated thematic analysis—qualitative data from all three phases were combined for analysis. The iterative process of analysis included writing post-interview reflections and thematic notes, conducting transcription quality assurance, identifying initial themes, recoding of transcripts after codebook refinement, and extracting quotes for the framework matrix. The framework approach to data analysis [55] was conducted using Microsoft Excel, allowing within- and between-participant analyses of quotations arranged by theme. Representative quotes of the resultant major themes were then selected to illustrate the findings.

Data triangulation occurred through immersion during the three-phase project with concurrent and ongoing data analysis. Specifically, a literature review and Phase 1 interviews were used to identify initial constructs, which were then used to develop survey items. We subsequently tested these survey items in Phase 2 using the cognitive interviewing procedure. Interview data from all Phases was used to guide the development of our hypothesized theoretical framework, which was tested using structural equation modeling and supported by qualitative data to provide nuanced data interpretation.

Quantitative, sample data were prepared in STATA 14.2 (Intercooled), factor analyses conducted in SPSS version 24 using principle axis factoring with oblique promax rotation, and structural equation modeling analyzed in MPlus version 8.2 using a maximum likelihood estimator and an added command to test indirect effects. Factor analyses were completed to ensure unidimensional construct measurement for the newly-developed scale measures before consideration in the structural equation model. PDPT intentions and information were estimated as manifest variables because of single scale measurement. Motivation, social support, and behavioral skills were estimated as latent variables with multiple indicators. Information, motivation, and social support were allowed to covary, and we included direct pathways from each of these three constructs to behavioral skills and PDPT intentions. We assessed model fit using χ^2^ badness-of-fit index, root mean square error approximation (RMSEA), comparative fit index (CFI), and standardized root mean square residual (SRMR). We assumed good model fit when the χ^2^/df ratio was 3 or less, RMSEA < 0.10, CFI > 0.90, and SRMR < 0.10 based on sample size and model complexity [56–59].

## Results

Phase 3 survey data were screened for obvious satisficing [60] and partial participation, which resulted in the exclusion of 3 survey respondents’ data. The final analytic sample included 197 survey responses and 25 qualitative interviews from patients presenting for HIV/STI counseling and testing at a Midwestern STI clinic. Of the 197 survey respondents, 61% were male. Most (91%) were Black or African American and 93% reported non-Hispanic/Latinx ethnicity. Mean age of the sample was 31.3 years (S.D. = 11.5; range: 18–62), and 41% of the sample had some college education or more. Nearly half (47%) were unemployed, 11% were uninsured, and 54% reported Medicaid/Medicare insurance coverage. Among those who participated in the qualitative components of the study, average patient age was 35.6 years. Most (84%) were Black or African American, and 14 men and 11 women were interviewed.

**Fig 1:**
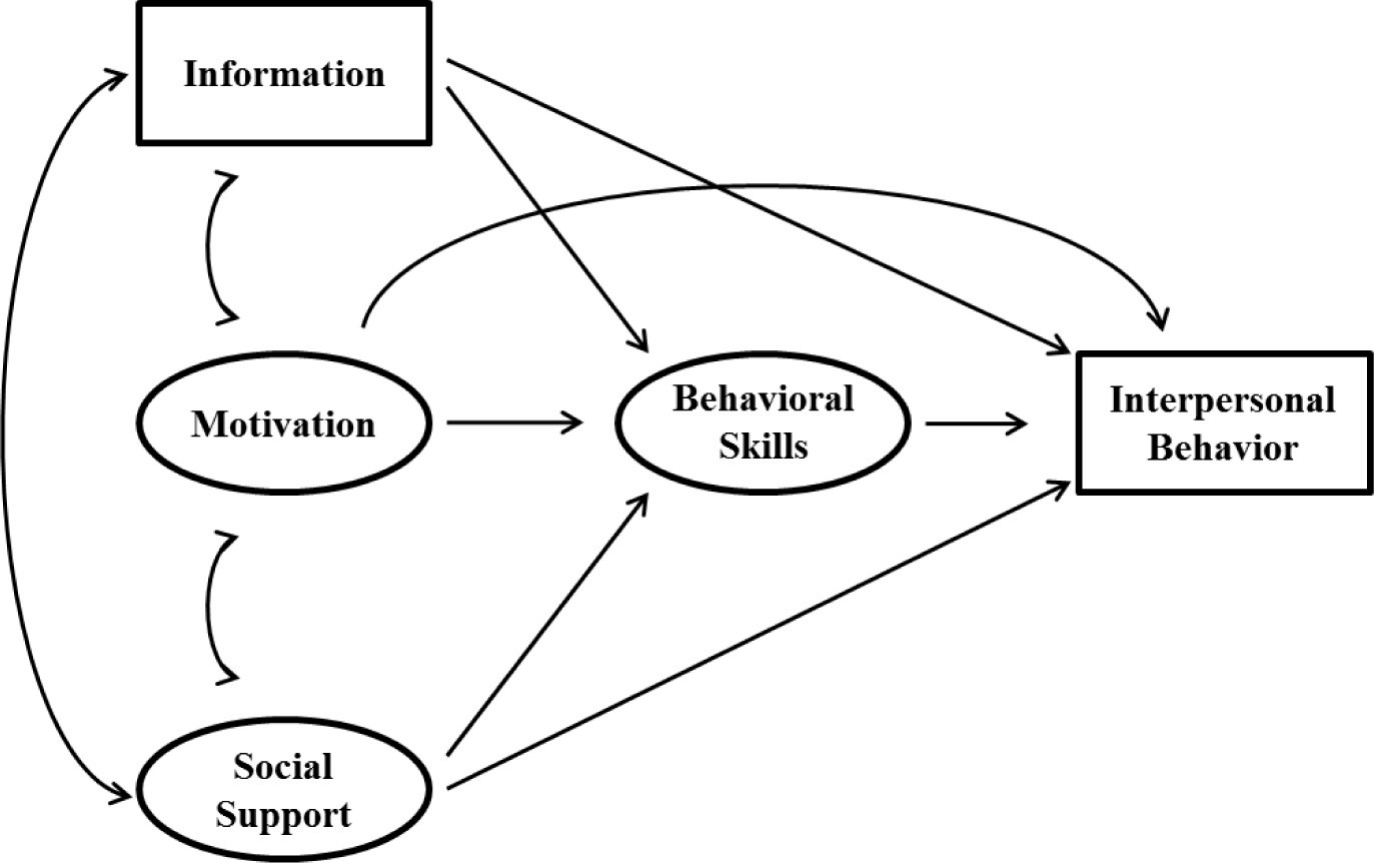
The Interpersonal-Behavior (I-B) model.

**Fig 2:**
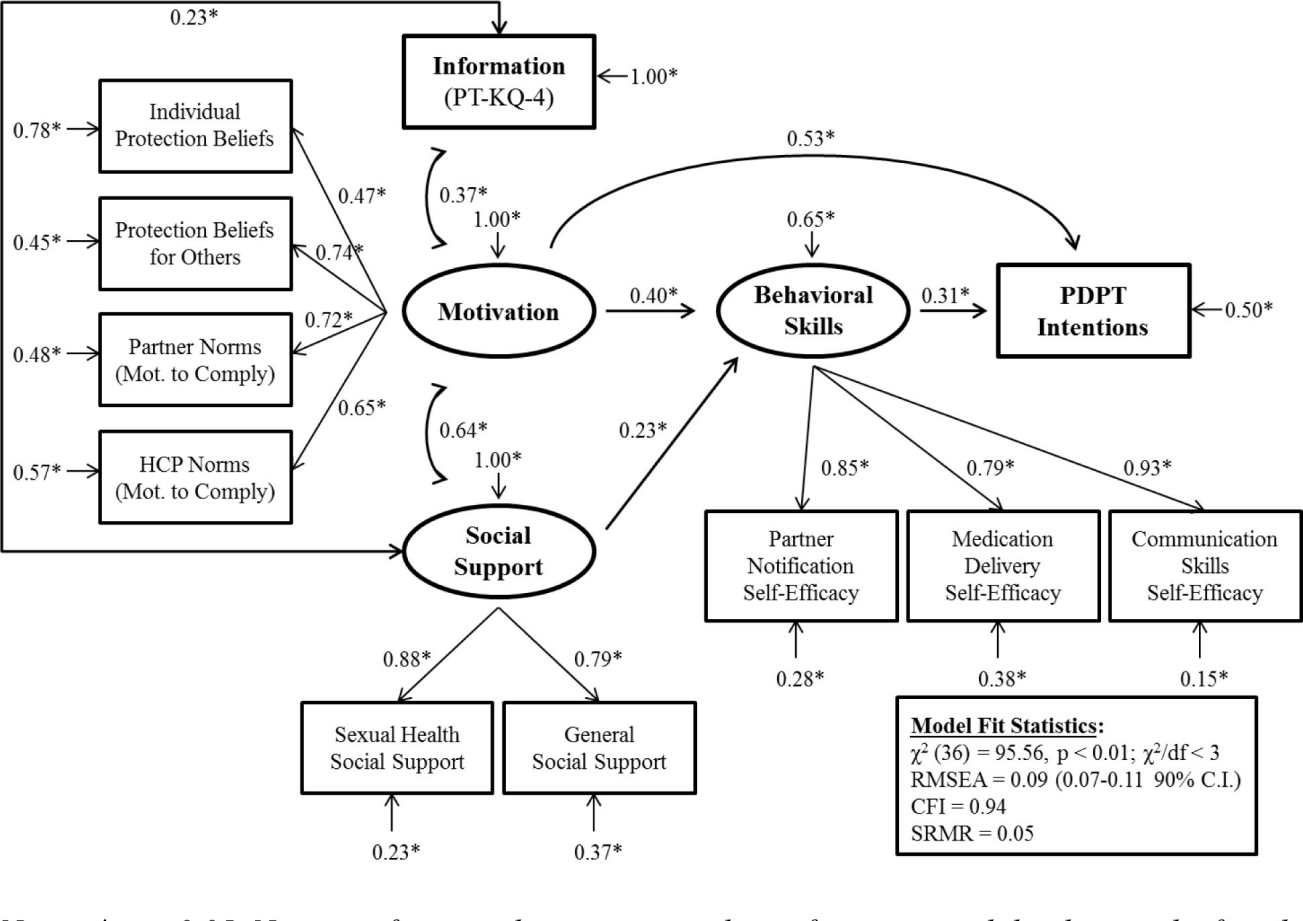
The structural equation model results testing the Interpersonal-Behavior model on intentions to use PDPT among STI clinic patients. *Notes*: * *p* < 0.05. Non-significant pathways are not shown for interpretability but can be found in the Results section.

Triangulation of qualitative and quantitative data resulted in the identification of key themes and a conceptual model explaining intentions to use PDPT. The conceptual model for understanding potential PDPT use, which we’ve titled the Interpersonal-Behavior (I-B) model, is illustrated in Fig 1. Descriptive statistics and scale reliabilities (i.e., Cronbach’s alpha) are presented in Table 1. Variances, covariances, and correlations of standardized indicator variables of the model are presented in Table 2. When a structural equation model was tested (Fig 2), the I-B model had good model fit [χ^2^ (36) = 95.56, *p* < 0.001; RMSEA = 0.09 (0.07–0.11 90% C.I.); CFI = 0.94; SRMR = 0.05] and explained 49.7% of the variance in PDPT intentions.

**Table 1:** Descriptive statistics and reliabilities of model indicators.

Indicator	Mean	SD	Range	α^a^
PT-KQ-4	3.32	1.15	0–4	0.77
Individual protection beliefs	4.53	0.73	1–5	0.62
Protection beliefs for others	4.20	0.79	1–5	0.79
Motivation-to-comply with partner norms	4.07	0.83	1–5	0.73
Motivation-to-comply with healthcare provider norms	4.20	0.78	1–5	0.81
Sexual health social support	4.02	0.98	1–5	0.88
General social support	3.96	1.16	1–5	0.95
Partner notification self-efficacy	4.44	0.75	1–5	0.87
Medication delivery self-efficacy	4.21	0.91	1–5	0.81
Communication skills self-efficacy	4.38	0.76	1–5	0.89
PDPT intentions	4.31	0.80	1–5	0.89

PT-KQ-4 = 4-item partner treatment knowledge questionnaire; PDPT = patient-delivered partner therapy

^a^ Reliability measured using Cronbach’s alpha.

**Table 2:** Variances, covariances, and correlations of indicator variables.

Indicator	1	2	3	4	5	6	7	8	9	10	11
1. PT-KQ-4	1.00	*0*.*27*	*0*.*36*	*0*.*18*	*0*.*17*	*0*.*22*	*0*.*16*	*0*.*31*	*0*.*13*	*0*.*20*	*0*.*30*
2. Individual protection beliefs	0.27	1.00	*0*.*45*	*0*.*25*	*0*.*27*	*0*.*27*	*0*.*18*	*0*.*20*	*0*.*17*	*0*.*18*	*0*.*30*
3. Protection beliefs for others	0.36	0.45	1.00	*0*.*51*	*0*.*46*	*0*.*39*	*0*.*45*	*0*.*33*	*0*.*25*	*0*.*36*	*0*.*46*
4. Motivation-to-comply with partner norms	0.18	0.25	0.50	1.00	*0*.*53*	*0*.*49*	*0*.*38*	*0*.*36*	*0*.*45*	*0*.*42*	*0*.*45*
5. Motivation-to-comply with healthcare provider norms	0.17	0.27	0.46	0.53	1.00	*0*.*23*	*0*.*32*	*0*.*29*	*0*.*37*	*0*.*34*	*0*.*50*
6. Sexual health social support	0.22	0.18	0.45	0.49	0.23	1.00	*0*.*69*	*0*.*37*	*0*.*30*	*0*.*43*	*0*.*32*
7. General social support	0.16	0.27	0.38	0.37	0.32	0.69	1.00	*0*.*30*	*0*.*27*	*0*.*37*	*0*.*37*
8. Partner notification self-efficacy	0.31	0.20	0.33	0.36	0.36	0.37	0.30	1.00	*0*.*67*	*0*.*79*	*0*.*48*
9. Medication delivery self-efficacy	0.13	0.17	0.25	0.45	0.45	0.30	0.27	0.67	1.00	*0*.*73*	*0*.*49*
10. Communication skills self-efficacy	0.20	0.18	0.36	0.42	0.42	0.43	0.37	0.79	0.73	1.00	*0*.*52*
11. PDPT intentions	0.30	0.30	0.45	0.45	0.50	0.32	0.37	0.48	0.49	0.51	1.00

Covariances in lower left, variances along diagonal, and correlations in upper right italicized; all covariances and variances were standardized.

The hypothesized associations between information, motivation, and social support in the conceptual model could best be described from a 48-year-old Black man: “*I've got a lot of support friends*. *All I have to do is call them and tell them what I'm going through and*, *information will be flying my way*. *‘Hey*, *you'll get over it*. *Maybe you should try this*, *or -*.*’ … You can always go to your guys and talk to them about sexual issues and stuff*.*”* This quote provides rationale for how social support members could have a role in motivating someone to engage in PDPT use, while also being a source of knowledge and advice. Similarly, patients described the need for more information about STIs, medications, and steps to resuming sexual activity. A 24-year-old Black woman described it here: “*You have to have the knowledge about the STD and the medication itself so that you’re ready answer to any questions that [your partners] may have for you*.*”* This woman’s quote provides specific informational needs of patients using PDPT.

Motivation was hypothesized to be associated with PDPT intentions and mediated by behavioral skills. This hypothesis was based on a breadth of discussion with patients about motivating factors that could influence PDPT use. A 29-year-old Black man described how he cared for the health of others: *“I [wouldn’t want] the partner having sexual intercourse with somebody else and giving them something too*.*”* Protection beliefs for others was balanced with individual protection beliefs, as mentioned by a 24-year-old Black woman: *“We want to get rid of [the STI] and be able to be comfortable and safe and know that OK*, *well*, *if we're both clean then if we do decide to have unprotected sex we don't have to have this problem pop up [again] or anything like that*.*”* Additionally, potential PDPT use was influenced by patients’ opinions about what their provider and partner would want them to do. As a 21-year-old Black man stated: *“I think that [providers] would feel like this*, *[PDPT is] a good thing to do*. *… They got a good opinion on it*, *so it [would] influence mine in a positive way*.*”*

Social support was hypothesized to be associated with behavioral skills, with behavioral skills mediating the relationship between social support and PDPT intentions. A 21-year-old Black woman described how social support affects one’s confidence (i.e., self-efficacy): *“If they had a lot of people in their ear*, *‘like you need to tell them*,*’ then that would kind of like boost their confidence about it*.*”* Although confidence could be influenced by social support members, specific skill needs were apparent. A 25-year-old Black man described the need for communication skills and the ability to answer questions about the infection and medication: *“Basic communication skills*. *Know how to talk to them and know how to say the right things… Knowledge of the situation*, *the medicine*, *knowing what you’re gonna say*. *Not only what you’re gonna say*, *but how you’re gonna say it*.*”* These objective skill needs were complimented by the need to increase the confidence of the patient through skills-based trainings. Specifically, a 59-year-old White man made the following statement: *“[You need to] build up the confidence of the person delivering the med*. *… So you've got to educate them*, *kind of train them on how to go about it*.*”* Collectively, these quotes provide context to our modeling results described next.

Structural equation model results are illustrated in Fig 2. Information was significantly associated with both motivation (*β* = 0.37, *p* < 0.001) and social support (*β* = 0.23, *p* = 0.002) but not behavioral skills (*β* = 0.04, *p* = 0.577) or PDPT intentions (*β* = 0.05, *p* = 0.391). Motivation was associated with social support (*β* = 0.64, *p* < 0.001) and behavioral skills (*β* = 0.40, *p* < 0.001), and social support was associated with behavioral skills (*β* = 0.23, *p* = 0.025). Behavioral skills were associated with higher PDPT intentions (*β* = 0.31, *p* < 0.001), partially mediated the association of motivation with intentions (*β*_direct_ = 0.53, *p* < 0.001; *β*_indirect_ = 0.12, 95% CI: 0.03–0.30), and fully mediated the association of social support with intentions (*β*_direct_ = -0.11, *p* = 0.253; *β*_indirect_ = 0.07, 95% CI: 0.00–0.21).

## Discussion

In this study, STI clinic patients discussed the influence of information, motivational factors, social support, and behavioral skills on hypothetical PDPT use. This qualitative data was then supported by structural equation modeling of the I-B model. Our work suggests an expansion of prior theories to incorporate both social support and interpersonal motivations as the best basis for future interventions targeting PDPT uptake.

We illustrate our proposed model, which we conceptualized based on an adaptation of the Informational-Motivation-Behavioral skills model [16–19] and other theories [61–64], in Fig 1. Although the study of the Information-Motivation-Behavioral skills model is robust and used to explain various HIV/STI prevention behaviors [20–48], research aimed at understanding intentions to use PDPT has been rather limited. Previous study of PDPT [6] was based on the Theory of Planned Behavior [61], incorporating attitudes, subjective norms, and perceived control. We expand on these prior findings to incorporate knowledge and self-efficacy, which were constructs similarly added to the Informational-Motivation-Behavioral skills [16–19] and Integrated Behavioral models [63]. Self-efficacy extends beyond perceived control in a mechanistic role for facilitating engagement in behavior when considered within volitional control. Further, we added social support based on participants’ reflections about the need for additional encouragement, knowledge, and advice. Uniquely, the I-B model combines these factors into a single model useful for understanding interpersonal sexual health related behaviors—the first known theoretical framework of its kind.

Although knowledge was an important construct identified in qualitative analyses, our quantitative modeling only found knowledge associated with motivation and social support. Information was retained as a construct of the I-B model because of the importance of knowledge facilitating motivation; lack of knowledge—such as the lack awareness for the potential of repeat infection without partner treatment, among other items included in the PT-KQ-4 scale measure—was associated with decreased motivation in our model. Moreover, lack of knowledge could be overcome by informational support from social network members, indicating the synergistic role of these constructs. Including the information construct was also supported by prior research, which found many STI clinic patients lacked an understanding of treatment, symptoms, and transmission concepts about bacteria and infections [7]. Thus, knowledge remains an important component of reducing repeat infection risk through partner treatment.

Motivational factors were critical, in line with prior research. Previous research indicated the importance of partner treatment verification as a facilitator to PDPT use [11, 15], and decreasing reinfection risk was perceived as important in research with patient-delivered STI screening kits [8]. Nonetheless, other studies with STI clinic patients found that patients do not always prioritize PDPT as a mechanism to reduce repeat infection; patients declined PDPT because of beliefs that treatment is unnecessary with condom use [14], further indicating the importance of knowledge and its connection to motivation in our analysis. As such, individual protection beliefs need to be supported with protection beliefs for others to ensure PDPT use. Some patients also indicated negative opinions about the partner believed to be responsible for the STI in our study, which decreased their motivation for providing them treatment as similarly identified previously [14]. Patients’ beliefs were also altruistic in our sample; patients had a desire to protect their partner’s health as similarly supported by prior research [7, 8, 11, 14]. Positive partner protection beliefs were identified as potential barriers to PDPT use; a desire for partners to receive face-to-face contact and counseling from a healthcare provider were identified as large barriers to PDPT use in prior research [13, 15]. Therefore, it is necessary to account for multiple forms of protection beliefs in the counseling of patients who receive PDPT.

Facilitating motivations for PDPT use were perceived normative beliefs about PDPT and partner notification. Patients discussed the importance of their and their partners’ health, but the appraisal of their partner’s opinion influenced their own about using PDPT, consistent with normative influences identified in prior research [14]. Motivation-to-comply with the normative influences of healthcare providers were also important; patients generally respected the opinions of their clinicians and support prior claims indicating the positive implications of healthcare providers’ support for PDPT on patient willingness to use it [6].

Social support offered an application to buffer negative life events and stressors. In prior research, individuals reported using social support members to facilitate partner notification and PDPT use [11, 14], indicating the potential instrumental support social network members may have in addition to the informational support identified by our participants. Social support is important because of the complexity to navigating partner notification and treatment delivery.

Behavioral skills fully mediated the relationship between social support and PDPT intentions, indicating social support might not be enough to influence PDPT use without supplemental, skill-based trainings. Patients stressed the need for communication skills training in another STI clinic sample [15], similar to our findings, and the need for more coaching with potential transcripts for how to discuss STIs with their partners as requested by patients previously [14]. Patients in our sample discussed the importance of developing a sense of confidence (i.e., self-efficacy) prior to partner notification and PDPT use, which was previously identified in the partner notification literature [12, 65]. However, no known behavioral interventions have rigorously developed and tested a skills-based training intervention to support PDPT use. Further research is needed to develop and test a comprehensive, supporting intervention for PDPT, incorporating the components of behavioral skills and the other I-B model constructs.

### Limitations

Several limitations of this research study merit mention. First, patients were interviewed and surveyed under a hypothetical scenario of being diagnosed with chlamydia at their current clinic visit; our results may not reflect actual behavioral intentions or behavior after an STI diagnosis. However, over two-thirds of patients in the study were diagnosed with an STI previously [49], and many participants discussed their current and prior diagnoses during interviews, suggesting the scenario was familiar to many. Second, the cross-sectional nature of our interviews and survey cannot rule out equivalent structural models. Further experimental and longitudinal research is needed to identify which constructs, and in what combinations, affect actual PDPT use. Third, results were based on a sample of patients recruited from a Midwestern STI clinic, potentially limiting generalizability. Further research is needed to determine the robustness of our findings with other patient populations.

## Conclusions

PDPT is an evidence-based method of partner treatment that would benefit from an accompanying behavioral intervention. STI clinic patients attending a Midwestern, public STI clinic discussed themes of information, motivation, social support, and behavioral skills as barriers and facilitators to PDPT use, which supported the development of a conceptual framework. Structural equation modeling of the Interpersonal-Behavioral (I-B) model to explain PDPT intentions had good model fit, supporting the hypothesized theoretical framework. Future intervention work should target I-B model constructs.
